# Chronic Alcohol Ingestion Increases Mortality and Organ Injury in a Murine Model of Septic Peritonitis

**DOI:** 10.1371/journal.pone.0062792

**Published:** 2013-05-22

**Authors:** Benyam P. Yoseph, Elise Breed, Christian E. Overgaard, Christina J. Ward, Zhe Liang, Maylene E. Wagener, Daniel R. Lexcen, Elizabeth R. Lusczek, Greg J. Beilman, Eileen M. Burd, Alton B. Farris, David M. Guidot, Michael Koval, Mandy L. Ford, Craig M. Coopersmith

**Affiliations:** 1 Department of Surgery and Emory Center for Critical Care, Emory University School of Medicine, Atlanta, Georgia, United States of America; 2 Department of Medicine and Emory Alcohol and Lung Biology Center, Emory University School of Medicine, Atlanta, Georgia, United States of America; 3 Department of Surgery and Emory Transplant Center, Emory University School of Medicine, Atlanta, Georgia, United States of America; 4 Department of Surgery, University of Minnesota, Minneapolis, Minnesota, United States of America; 5 Department of Pathology and Laboratory Medicine, Emory University School of Medicine, Atlanta, Georgia, United States of America; University of Cincinnati, United States of America

## Abstract

**Background:**

Patients admitted to the intensive care unit with alcohol use disorders have increased morbidity and mortality. The purpose of this study was to determine how chronic alcohol ingestion alters the host response to sepsis in mice.

**Methods:**

Mice were randomized to receive either alcohol or water for 12 weeks and then subjected to cecal ligation and puncture. Mice were sacrificed 24 hours post-operatively or followed seven days for survival.

**Results:**

Septic alcohol-fed mice had a significantly higher mortality than septic water-fed mice (74% vs. 41%, p = 0.01). This was associated with worsened gut integrity in alcohol-fed mice with elevated intestinal epithelial apoptosis, decreased crypt proliferation and shortened villus length. Further, alcohol-fed mice had higher intestinal permeability with decreased ZO-1 and occludin protein expression in the intestinal tight junction. The frequency of splenic and bone marrow CD4+ T cells was similar between groups; however, splenic CD4+ T cells in septic alcohol-fed mice had a marked increase in both TNF and IFN-γ production following *ex vivo* stimulation. Neither the frequency nor function of CD8+ T cells differed between alcohol-fed and water-fed septic mice. NK cells were decreased in both the spleen and bone marrow of alcohol-fed septic mice. Pulmonary myeloperoxidase levels and BAL levels of G-CSF and TFG-β were higher in alcohol-fed mice. Pancreatic metabolomics demonstrated increased acetate, adenosine, xanthine, acetoacetate, 3-hydroxybutyrate and betaine in alcohol-fed mice and decreased cytidine, uracil, fumarate, creatine phosphate, creatine, and choline. Serum and peritoneal cytokines were generally similar between alcohol-fed and water-fed mice, and there were no differences in bacteremia, lung wet to dry weight, or pulmonary, liver or splenic histology.

**Conclusions:**

When subjected to the same septic insult, mice with chronic alcohol ingestion have increased mortality. Alterations in intestinal integrity, the host immune response, and pancreatic metabolomics may help explain this differential response.

## Introduction

Alcohol use disorders represent a serious challenge to public health. Alcohol abuse and dependence affects over 75 million people worldwide and accounts for 2.5 million deaths/year [1]. In the United States, 18.3 million people have alcohol use disorders [2]. An estimated 20–40% of patients admitted to the hospital have alcohol use disorders, and up to one third of patients admitted to the intensive care unit (ICU) have alcohol use disorders [2]–[6].

Alcohol use disorders are associated with significant morbidity and mortality in critically ill patients. Multiple studies have shown patients with alcohol use disorders have longer ICU stays, more frequent admissions to the ICU, and an increased risk of death [7]–[9]. Additionally, patients with chronic alcohol abuse with septic shock have an increased incidence of acute respiratory distress syndrome and severity of multiple organ dysfunction levels compared to those without a history of alcohol abuse [10], [11]. Surgical patients with a history of chronic alcohol abuse have increased complications including sepsis and need for repeat surgery [12]–[14]. Additionally, patients in the community with chronic alcohol abuse have a higher likelihood of developing sepsis and higher rates of developing sepsis when they become hospitalized, associated with longer lengths of stay and higher hospital charges [15]–[18].

In isolation, both chronic alcohol abuse and sepsis cause widespread damage on a cellular and tissue level. There is, however, relatively little understood regarding how chronic alcohol ingestion affects a host that subsequently becomes septic. It has recently been shown that increasing alcohol concentration leads to elevated mortality in a rat model of polymicrobial peritonitis induced by intraperitoneal fecal injection, and this is associated with decreased IL-6 and TNF in alcohol-fed animals [19]. Additionally, rats subjected to cecal ligation and puncture (CLP, a model of peritonitis) following chronic alcohol ingestion have lower glutathione levels and fluid protein levels in lung lavage fluid as well as worsened hypoxemia [20]. To better understand potential mechanisms of how alcohol use disorders alter the host response to sepsis, we examined mice subjected to CLP following chronic alcohol ingestion.

## Materials and Methods

### Animals and chronic alcohol ingestion model

Six week old male FVB/N mice were obtained from Charles River Laboratories (Wilmington, MA). After an acclimatization period of one week, mice were randomized to receive either alcohol or water. Mice were acclimated to alcohol by increasing its concentration from 0% to 20% (weight/volume) over the course of two weeks (5% w/v for 4 days, 10% w/v for 4 days, 15% w/v for 4 days). Following this, animals received 20% concentration of alcohol for an additional ten weeks [21], [22]. Control animals drank water during this same time period. All animals had access to laboratory chow *ad libitum* throughout. All experiments were performed in accordance with the National Institutes of Health Guidelines for the Use of Laboratory Animals and were approved by the Institutional Animal Care and Use Committee at Emory University School of Medicine (Protocol DAR-2001256-110214BN). All surgery (described below) was performed under isoflurane anesthesia and all efforts were made to minimize animal suffering. Specifically, all animals were given a single dose of buprenex post-operatively and as deemed appropriate thereafter by the veterinary staff at Emory University.

### Sepsis model

After receiving twelve weeks of alcohol or water, a subset of mice were subjected to CLP according to the method of Baker et al. [23]. Under isoflurane anesthesia, a small midline abdominal incision was made, and the cecum was ligated just distal to the ileocecal valve using a technique that did not result in intestinal obstruction. The cecum was then punctured twice with a 25-gauge needle and gently squeezed to extrude a small amount of stool. After its contents were returned to the abdominal cavity, the incision was closed in layers. All septic mice were injected subcutaneously with 1 ml of normal saline to account for insensible fluid losses that occurred during surgery. Animals were euthanized at either 24 hours for functional studies or followed 7 days for survival. Animals received either two doses of antibiotics (ceftriaxone 25mg/kg + metronidazole 12.5 mg/kg, intraperitoneally) at 3 and 15 hours postoperatively or 48 hours of antibiotics depending on whether they were being euthanized at 24 hours or followed for survival.

### Intestinal epithelial apoptosis

Apoptotic cells in the intestinal epithelium were quantified by H&E-staining and active caspase-3 staining in 100 contiguous well-oriented crypt-villus units [24], [25]. Apoptotic cells were identified on H&E-stained sections using morphologic criteria, which identified cells with characteristic nuclear condensation and fragmentation. For active caspase-3 staining, jejunal sections were deparaffinized, rehydrated, and incubated in 3% hydrogen peroxide for 10 minutes. Slides were then immersed in Antigen Decloaker (Biocare Medical, Concord, CA) and heated in a pressure cooker for 45 minutes to facilitate antigen retrieval. Sections were then blocked with 20% goat serum (Vector Laboratories, Burlingame, CA) and incubated with rabbit polyclonal anti-active caspase-3 (1:100; Cell Signaling Technology, Beverly, MA) overnight at 4°C. They were then incubated with goat anti-rabbit biotinylated secondary antibody (1:200; Vector Laboratories) for 30 minutes at room temperature, followed by Vectastain Elite ABC reagent (Vector Laboratories) for 30 minutes at room temperature and developed with diaminobenzidine followed by counterstaining with hematoxylin.

### Villus length

Villus length was measured on H&E-stained sections as the distance in μm from the crypt neck to the villus tip in 12 well-oriented jejunal villi per animal using Image J software (National Institutes of Health, Bethesda, MD).

### Intestinal proliferation

Animals received an intraperitoneal injection of 5-bromo-2′deoxyuridine (BrdU, 5 mg/mL diluted in normal saline; Sigma, St. Louis, MO) 90 min prior to sacrifice to label cells in S-phase. Intestinal sections were deparaffinized, rehydrated, incubated in 1% hydrogen peroxide, immersed in Antigen Decloaker and heated in a pressure cooker for 45 minutes. Sections were then blocked for 10 min with Protein Block (Dako, Carpinteria, CA) and incubated with rat monoclonal anti-BrdU (1:500; Accurate Chemical & Scientific, Westbury, NY) overnight at 4°C. After being incubated at room temperature with goat anti-rat secondary antibody (1:500; Accurate Chemical & Scientific) for 30 minutes, sections were incubated with streptavidin-horseradish peroxidase (1:500; Dako) for 60 minutes and developed with diaminobenzidine, followed by counterstaining with hematoxylin. S-phase cells were quantified in 100 contiguous crypts.

### Intestinal permeability

Intestinal permeability was measured *in vivo* to fluorescein isothiocyanate conjugated-dextran (FD-4, 22mg/ml, molecular mass 4.4 kDa) [26]. Five hours before sacrifice, animals were gavaged with 0.5 ml of FD-4. At time of sacrifice, blood was collected and centrifuged at 3000 rpm at 4°C for 20 minutes. Plasma (50 μl) was then diluted with an equal amount of sterile phosphate-buffered saline (pH 7.4), and the concentration of FD-4 was determined using fluorospectrometry (NanoDrop 3300, Thermo Scientific, Wilmington, DE) using an excitation wavelength of 470 nm and an emission wavelength of 515 nm with serially diluted samples as standards. All samples and standards were run in triplicate.

### Tight junction expression

Protein expression of claudin-2, occludin, and ZO-1 was evaluated by Western blot. Frozen segments of jejunum were homogenized in 5x volume of ice-cold homogenization buffer and centrifuged at 10,000 rpm at 4°C for 5 minutes [27], [28]. The supernatant was collected, and total protein concentration was determined via the Bradford protein assay. Protein samples of 40 µg and equal volume of 2x Laemmli buffer were heated at 95°C for 5 minutes. Samples were run on polyacrylamide gels (Bio-Rad, Hercules, CA) and then transferred to Immuno-Blot polyvinylidenedifluoride membrane for 2 hours at 80V. Membranes were then blocked in 5% nonfat milk in Tris-buffered saline with 0.1% Tween 20 (Sigma) at room temperature for 60 minutes and incubated overnight with primary antibody in 4°C. The following primary antibodies were used: rabbit anti-claudin-2 (AbcamInc, Cambridge, MA), rabbit anti-occludin, rabbit anti-ZO-1, and rabbit anti-β-actin(Cell Signaling Technology). Membranes were then washed and incubated for 60 minutes at room temperature with horseradish peroxidase-conjugated goat anti-rabbit (Cell Signaling Technology) and developed with a chemiluminescent system (Pierce, Rockford, IL) after exposure to x-ray film.

### Flow cytometry and intracellular cytokine staining

Splenocytes and bone marrow cells from femur and tibia were harvested, processed into single cells suspensions and stained with CD4-Pacific Blue, CD8a-APC, and NK1.1 PE (BD Bioscience, San Jose, CA) for flow cytometric analysis on a LSRII flow cytometer (BD Biosciences). Data were analyzed using FlowJo Software (Tree Star, San Carlos, CA).

To measure production of IFN-γ, TNF, and IL-2 on a per cell basis, splenocytes were stimulated with phorbol 12-myristate 13-acetate (PMA, 30 ng/mL) and ionomycin (400 ng/mL) in the presence of 10μg/mL of Brefeldin A. After 18 hours, cells were surface stained with anti-CD4 and anti-CD8 as described above and processed with an intracellular staining kit (BD Biosciences) according to manufacturer's instructions. Intracellular antibodies included anti-IFN-γ (eBioscience, San Diego, CA), anti-TNF, and anti-IL-2 (both BD Biosciences). Data were acquired on a LSR II flow cytometer and analyzed using FlowJo Software.

### Lung histology, weights and myeloperoxidase (MPO) activity

H&E-stained lung sections were evaluated for the presence of histopathology by an observer blinded to tissue identity [22]. In a separate set of animals, the left lung was excised and weighed to obtain a “wet” weight. Lungs were then dried for 16 hours in an oven at 115°C and reweighed to establish a dry weight. To quantify MPO, lungs were homogenized and proteins resolved by SDS-PAGE. MPO was detected by immunoblot using rabbit anti-MPO (Sigma) using enhanced chemiluminescence reagent (GE Healthcare, Pittsburgh, PA). Blots were imaged and quantified using a ChemiDoc XRS+ Molecular Imaging System (BioRad) and analyzed using Image Lab 2.0.1 software [29].

### Systemic and local cytokines

Bronchoalveolar lavage (BAL) fluid was obtained by cannulating the trachea with a 22-gauge angiocatheter, and lavaging the lungs with 1 ml of PBS. Peritoneal fluid was obtained by lavaging the abdominal cavity with 3 ml of PBS. BAL, peritoneal fluid and blood samples from each mouse were centrifuged for 5 minutes at 10,000g, and cytokine concentrations were then evaluated using a multiplex cytokine assay (Bio-Rad) according to manufacturer's instructions. The lower detection limits for the measured cytokines in pg/ml were as follows: IL-1β 2.94, IL-6 0.74, IL-10 0.73, IL-13 1.54, G-CSF 3.02, IFN-γ 1.45, MCP-1 1.37, TNF-α 2.15. All samples were run in duplicate.

### Cultures

Serum and peritoneal fluid samples were serially diluted in sterile 0.9% saline and plated on sheep blood agar plates. Colony counts were enumerated after incubation in 5% carbon dioxide for 24 hours at 37°C.

### Histology

Spleen, liver and pancreas tissues were harvested when animals were sacrificed, fixed, embedded in paraffin, sectioned, and stained with H&E for light microscopy. Histological analysis was performed by a pathologist (ABF) blinded to group identity.

### ^1^H NMR metabolomics analysis of pancreatic tissue

Pancreatic water-soluble metabolites were extracted with a perchloric acid protocol [30]. The extracted, lyophilized metabolites were reconstituted in D_2_O (Sigma) with an added internal standard (0.25 mM 4,4-dimethyl-4-silapentane-1-sulfonic acid in D_2_O), and samples were transferred into 5 mm NMR tubes (Wilmad, LabGlass, Vineland, NJ). One dimensional ^1^H NMR spectra were acquired on a 700 MHz BrukerrAvance NMR spectrometer (Bruker, Billerica, MA) with a 5 mm TXI proton-enhanced CryoProbe. A Carr-Purcell-Meiboom-Gill presaturation pulse sequence was used to acquire all spectra with 128 scans, which were subsequently phase- and baseline-corrected. The spectra were used to identify and quantify 52 metabolites in each sample using Chenomx NMR Suite 7 (Edmonton, Alberta, Canada). Metabolite concentrations were derived from internal standard, and expressed in mM. Several spiking experiments were conducted to validate suspected metabolites identified in the spectra.

Metabolite concentrations were exported from Chenomx into R Software (Vienna, Austria) for further analysis [31]. Data was preprocessed using unit variance scaling to give equal weight to metabolites of low and high concentrations. After scaling, partial least squares discriminate analysis (PLS-DA) was used to reduce the dimensionality of the data, yielding a statistical model optimizing separation of the treatment groups according to metabolic profile. The model was cross-validated to prevent over-fitting.

### Statistics

Data were analyzed using the statistical software program Prism 5.0 (GraphPad San Diego, CA) and are presented as mean ± SEM. Survival studies were analyzed using the Log-Rank test. Multi-group comparisons were made by one-way ANOVA followed by the Tukey post-test. Two-way comparisons were tested for Gaussian distribution using the Shapiro-Wilk normality test. If data were found to have Gaussian distributions, comparisons were performed using the Student's t-test. If data did not have Gaussian distribution, two-way comparisons were performed using the Mann Whitney test. A p value of <0.05 was considered to be statistically significant.

## Results

### Effect of chronic alcohol ingestion on mortality from sepsis

**Figure 1:**
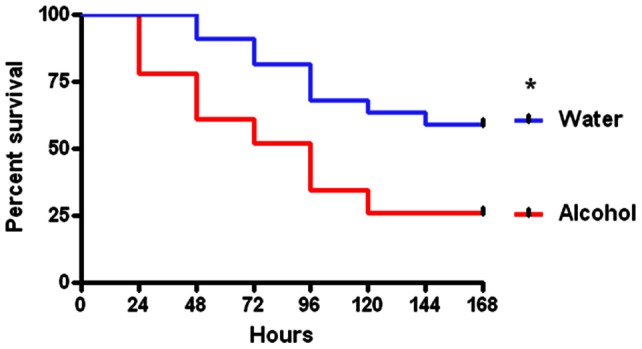
Effect of chronic alcohol ingestion on survival from sepsis. Alcohol-fed and water-fed mice (n = 22–23/group) were subjected to CLP. Septic alcohol-fed mice had significantly higher mortality than water-fed mice subjected to the same insult (p = 0.01).

In all experiments, comparisons were made between animals with chronic alcohol ingestion for 12 weeks prior to the onset of sepsis and control mice that were fed water during the same time period. Mice with chronic alcohol ingestion (n = 23) had a higher seven-day mortality following CLP than control mice (n = 22) subjected to the same insult (74% vs.41%, p = 0.01, Figure 1).

### Effect of chronic ethanol ingestion on gut integrity before and after sepsis

**Figure 2:**
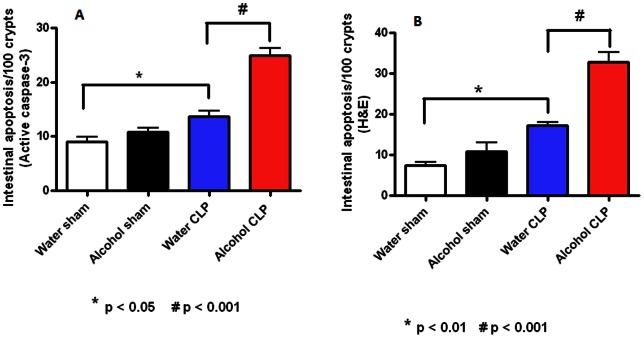
Effect of chronic alcohol ingestion on intestinal epithelial apoptosis. Chronic alcohol ingestion did not impact apoptosis in sham mice compared to water-fed mice (n = 8–10/group) whether assayed by active caspase 3 (A) or H&E (B). Septic water-fed mice (n = 8–10/group) had higher apoptosis than sham water-fed mice. Septic alcohol-fed mice (n = 9/group) had higher apoptosis than septic water-fed mice.

Intestinal epithelial apoptosis was similar in sham alcohol-fed and water-fed mice by both active caspase-3 and H&E and staining (Figure 2). Sepsis increased intestinal apoptosis in both alcohol-fed and water-fed animals compared to sham animals given alcohol or water respectively. Intestinal apoptosis was significantly higher in septic alcohol-fed mice than septic water-fed mice (25±3 vs. 14±4 cells/100 crypts, p = 0.0001 for active caspase 3, 32±7 vs. 17±2 cells/100 crypts, p = 0.0001 for H&E).

**Figure 3:**
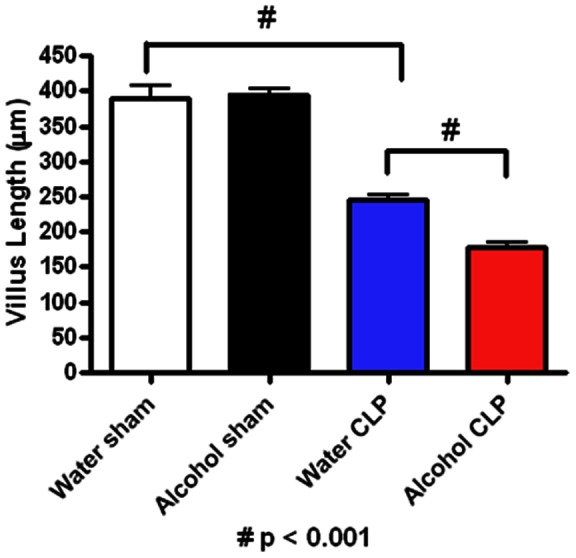
Effect of chronic alcohol ingestion on villus length. Chronic alcohol ingestion did not impact villus length in sham mice compared to water-fed mice (n = 9–10/group). Septic water-fed mice (n = 9/group) had shorter villi than sham water-fed mice. Septic alcohol-fed mice (n = 9/group) had a further diminution in villus length compared to septic water-fed mice.

Villus length was similar in sham alcohol-fed and water-fed mice (Figure 3). Sepsis decreased villus length in both alcohol-fed and water-fed animals compared to sham animals given alcohol or water respectively. Villus length was significantly shorter in septic alcohol-fed mice than septic water-fed mice (178±8 vs. 246±7 µm, p = 0.0002).

**Figure 4:**
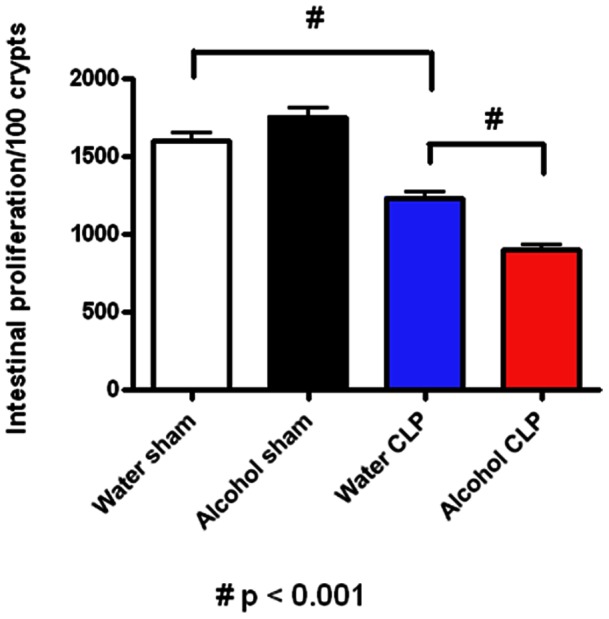
Effect of chronic alcohol ingestion on crypt proliferation. Chronic alcohol ingestion did not impact the number of BrdU positive cells in sham mice compared to water-fed mice (n = 5/group). Septic water-fed mice (n = 7/group) had fewer S-phase cells in the crypt than sham water-fed mice. Septic alcohol-fed mice (n = 6/group) had a further diminution in proliferating crypt cells compared to septic water-fed mice.

Intestinal proliferation was also similar in sham alcohol-fed and water-fed mice as measured by BrdU staining (Figure 4). Sepsis decreased proliferation in both alcohol-fed and water-fed animals compared to sham animals given alcohol or water respectively. Proliferation was lower in septic alcohol-fed mice than septic water-fed mice (1235±97 vs. 901±83 cells/100 crypts, p = 0.001).

### Effect of chronic ethanol ingestion on intestinal permeability before and after sepsis

**Figure 5:**
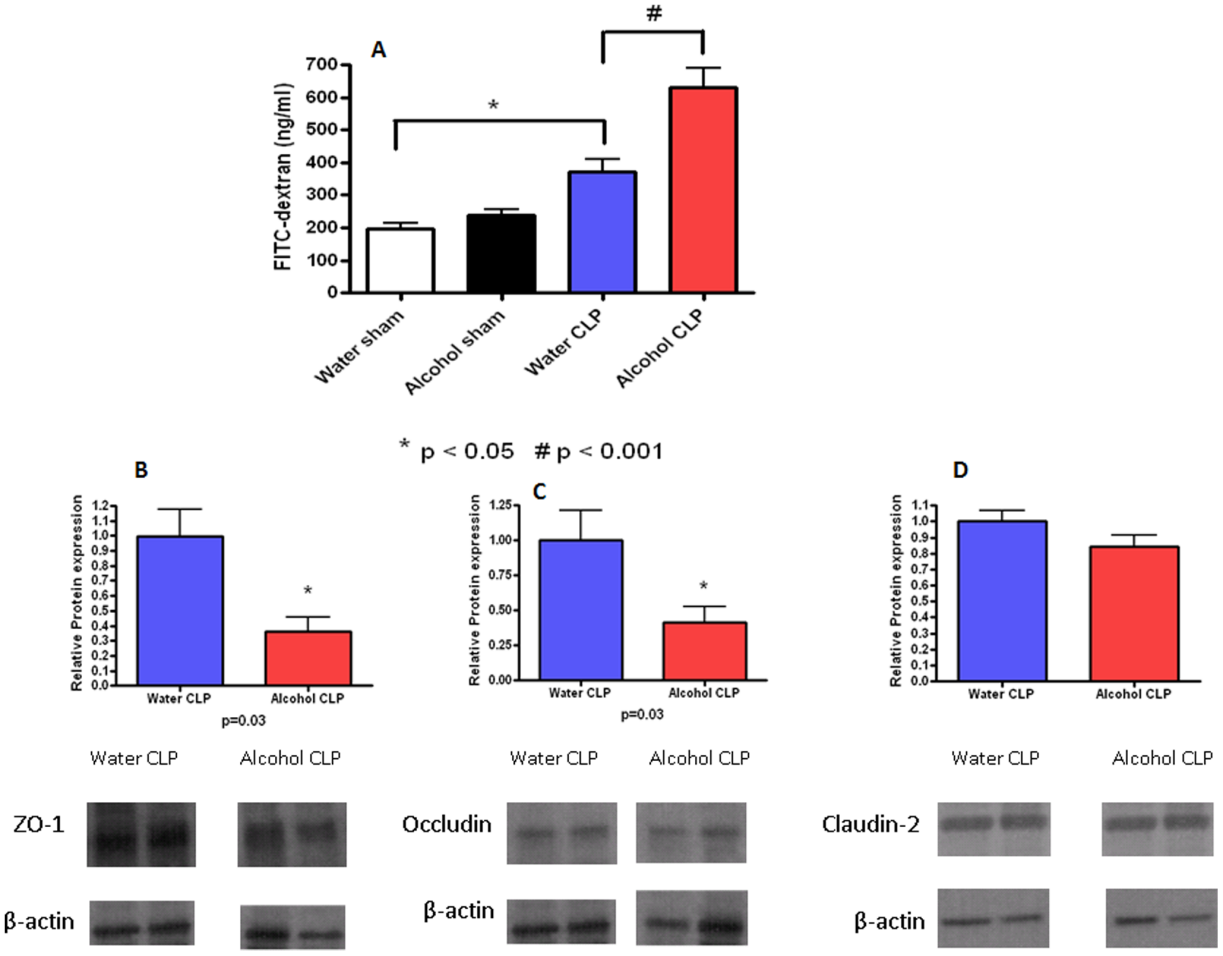
Effect of chronic alcohol ingestion on intestinal permeability. Chronic alcohol ingestion did not impact permeability (A) in sham mice compared to water-fed mice (n = 10/group). Septic water-fed mice (n = 13/group) had hyperpermeability compared to sham water-fed mice. Septic alcohol-fed mice (n = 15/group) had a further increase in intestinal permeability compared to septic water-fed mice. Protein levels of ZO-1 (B) and occludin (C) were lower in septic alcohol-fed mice compared to septic water-fed mice although claudin-2 levels (D) were similar. Representative blots for each tight junction protein are depicted; densitometry was determined by normalizing expression to β-actin.

Intestinal permeability was similar in sham alcohol-fed and water-fed mice (Figure 5A). Sepsis increased permeability in both alcohol-fed and water-fed animals compared to sham animals given alcohol or water respectively. Permeability was higher in septic alcohol-fed mice than septic water-fed mice as measured by the appearance of FD-4 in the bloodstream (632±57 vs. 372±38 pg/ml, p = 0.0009). To determine whether alterations in tight junction proteins contributed to intestinal barrier dysfunction in septic alcohol-fed vs. water-fed mice, ZO-1, occludin, and claudin-2 expression in the intestinal epithelium were evaluated by western blot (Figure 5B–C). Both ZO-1 and occludin were decreased in septic alcohol-fed mice compared to septic water-fed mice (p = 0.03, for both). In contrast, claudin-2 expression was similar in septic alcohol-fed mice and septic water-fed mice.

### Effect of chronic ethanol ingestion on immune response following sepsis

**Figure 6:**
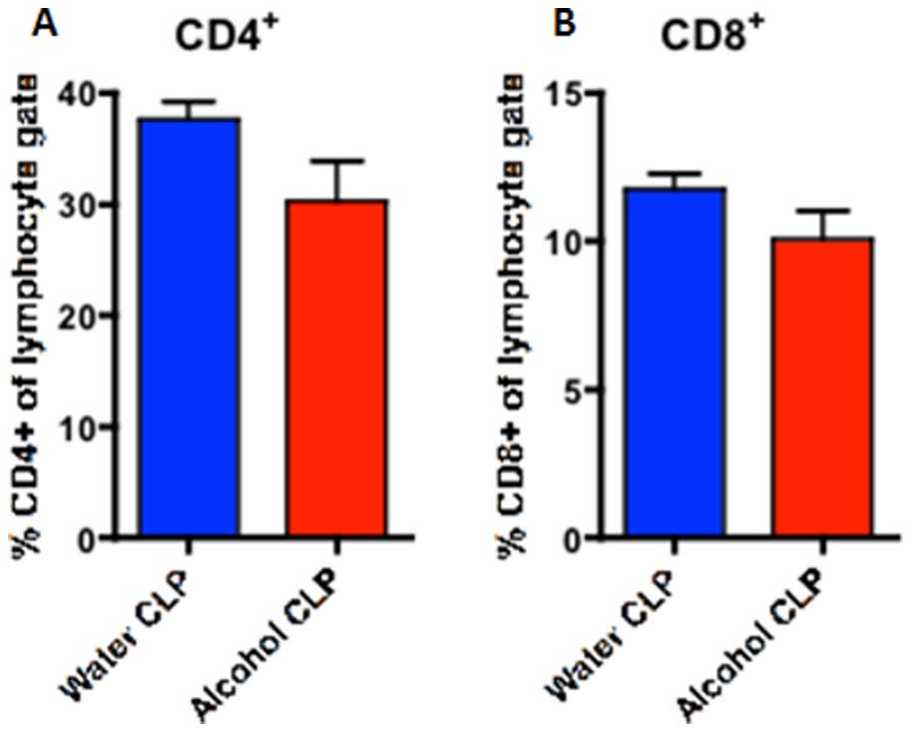
Effect of chronic alcohol ingestion on the size of CD4+ and CD8+ T cell compartments in the spleen during sepsis. Chronic alcohol ingestion did not impact frequencies of CD4+ and CD8+ T cells in the spleen during sepsis. Septic water-fed mice (n = 8/group) had similar frequencies of (A) CD4+ and (B) CD8+ T cells as compared to septic alcohol-fed mice (n = 8/group).

**Figure 7:**
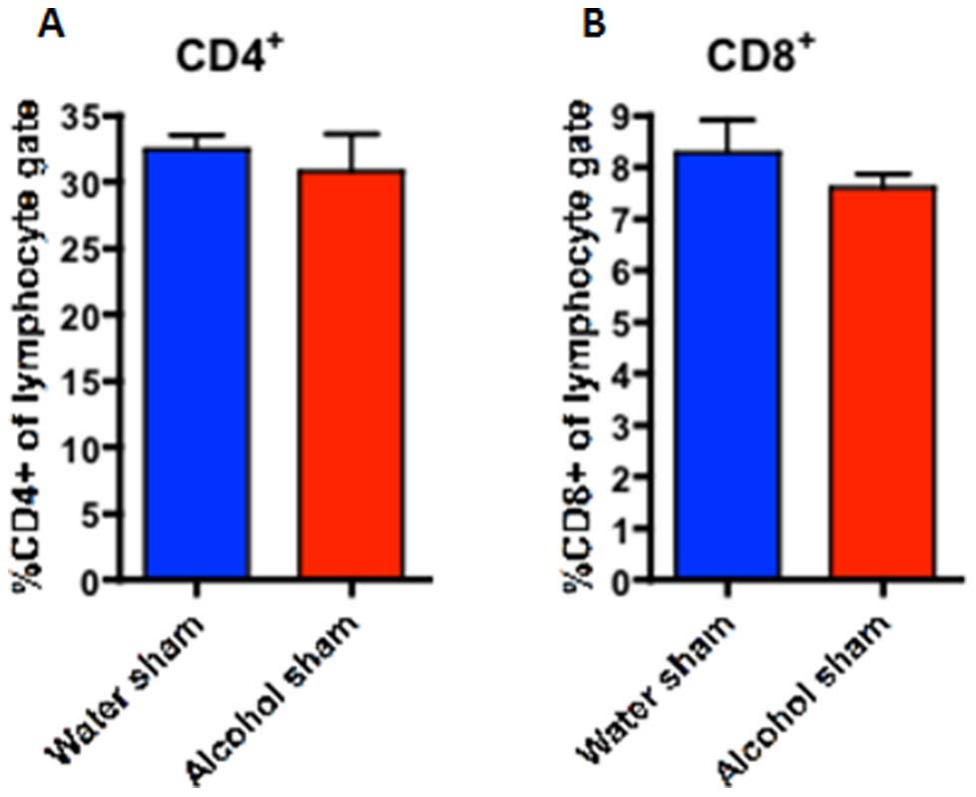
Effect of chronic alcohol ingestion on the size of CD4+ and CD8+ T cell compartments in the spleen in sham mice. Chronic alcohol ingestion did not impact frequencies of CD4+ and CD8+ T cells in the spleen in sham mice. Sham water-fed mice (n = 5/group) had similar frequencies of (A) CD4+ and (B) CD8+ T cells as compared to sham alcohol-fed mice (n = 5/group).

**Figure 8:**
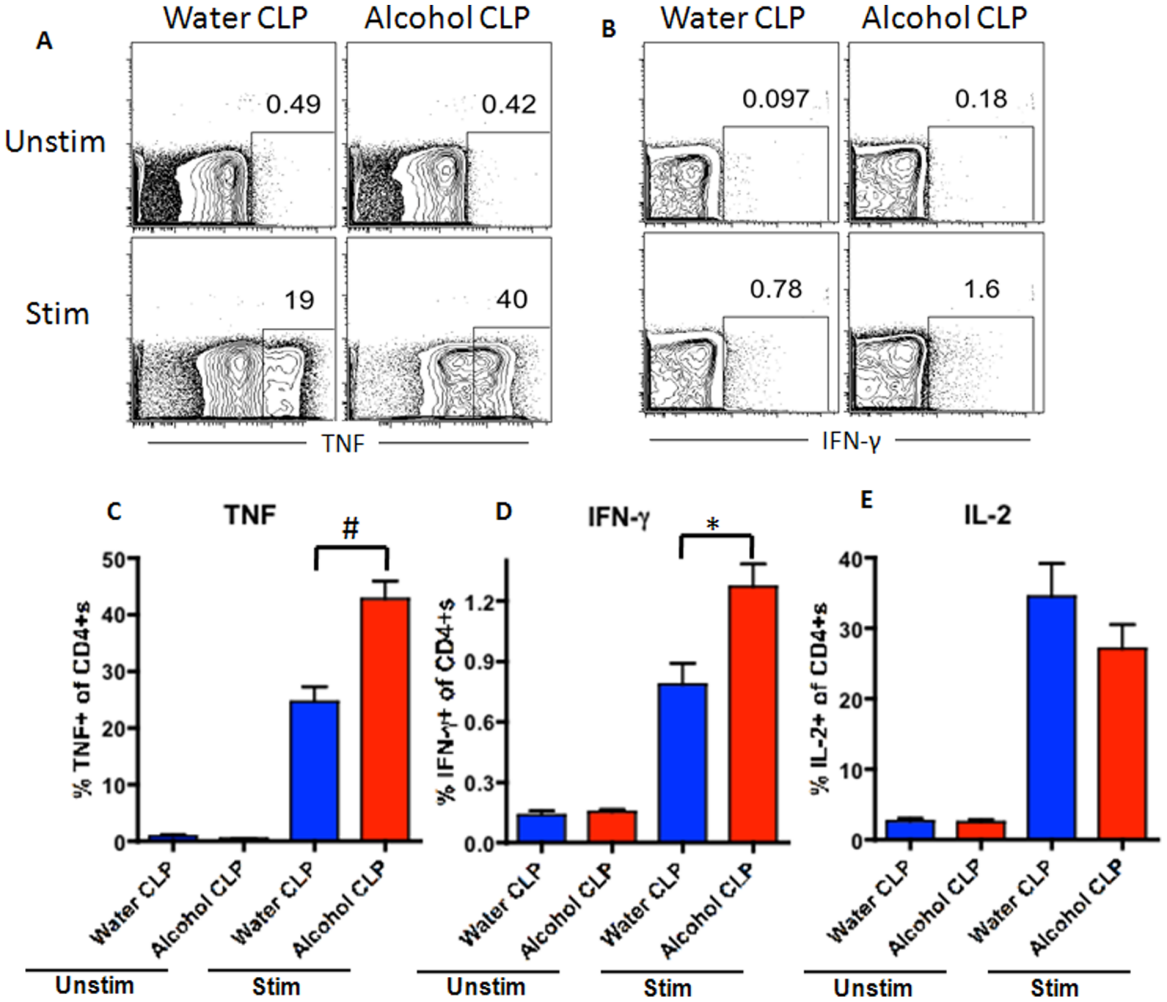
Effect of chronic alcohol ingestion on inflammatory cytokine secretion by CD4+ T cells during sepsis. Splenocytes from septic water-fed mice (n = 6/group) and septic alcohol-fed mice (n = 8/group) were harvested, restimulated with PMA/ionomycin for 18 h *ex vivo*, and stained intracellularly for the presence of inflammatory cytokines. The frequencies of TNF-secreting (A, C), and IFN-γ-secreting (B, D) CD4+ T cells were significantly increased in septic alcohol-fed mice as compared to septic water-fed mice. The frequency of IL-2-secreting CD4+ T cells (E) was unchanged between septic alcohol-fed mice and septic water-fed mice.

**Figure 9:**
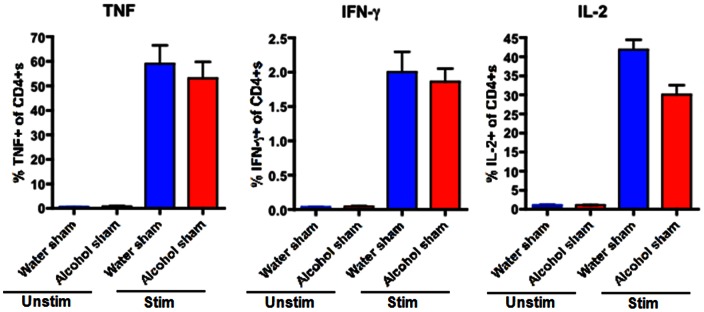
Effect of chronic alcohol ingestion on inflammatory cytokine secretion by CD4+ T cells in sham mice. Splenocytes from sham water-fed mice (n = 5/group) and sham alcohol-fed mice (n = 5/group) were harvested, restimulated with PMA/ionomycin for 18 h *ex vivo*, and stained intracellularly for the presence of inflammatory cytokines. The frequencies of TNF-secreting, IFN-γ-secreting, and IL-2-secreting CD4+ T cells were unchanged between sham alcohol-fed mice and sham water-fed mice.

CD4+ T cells comprised a similar frequency of total lymphocytes in the spleen of both septic alcohol-fed and septic water-fed mice (Figure 6A). This was also true for sham alcohol-fed and sham water-fed mice (Figure 7A). However, splenic CD4+T cells isolated from septic alcohol-fed mice exhibited a marked increase in both TNF (p = 0.0007) and IFN-γ (p = 0.003) production following *ex vivo* stimulation as compared to those isolated from septic water-fed mice (Figure 8A–D). No significant difference was noted in IL-2 production (Figure 8E). This increase in TNF and IFN-γ production was specific to the setting of sepsis, as no differences in the production of TNF or IFN-γ following *ex vivo* restimulation were noted in sham alcohol-fed mice as compared to sham water-fed mice (Figure 9). There was also no difference in the production of IL-2 between sham alcohol-fed mice and sham water-fed mice (Figure 9).

**Figure 10:**
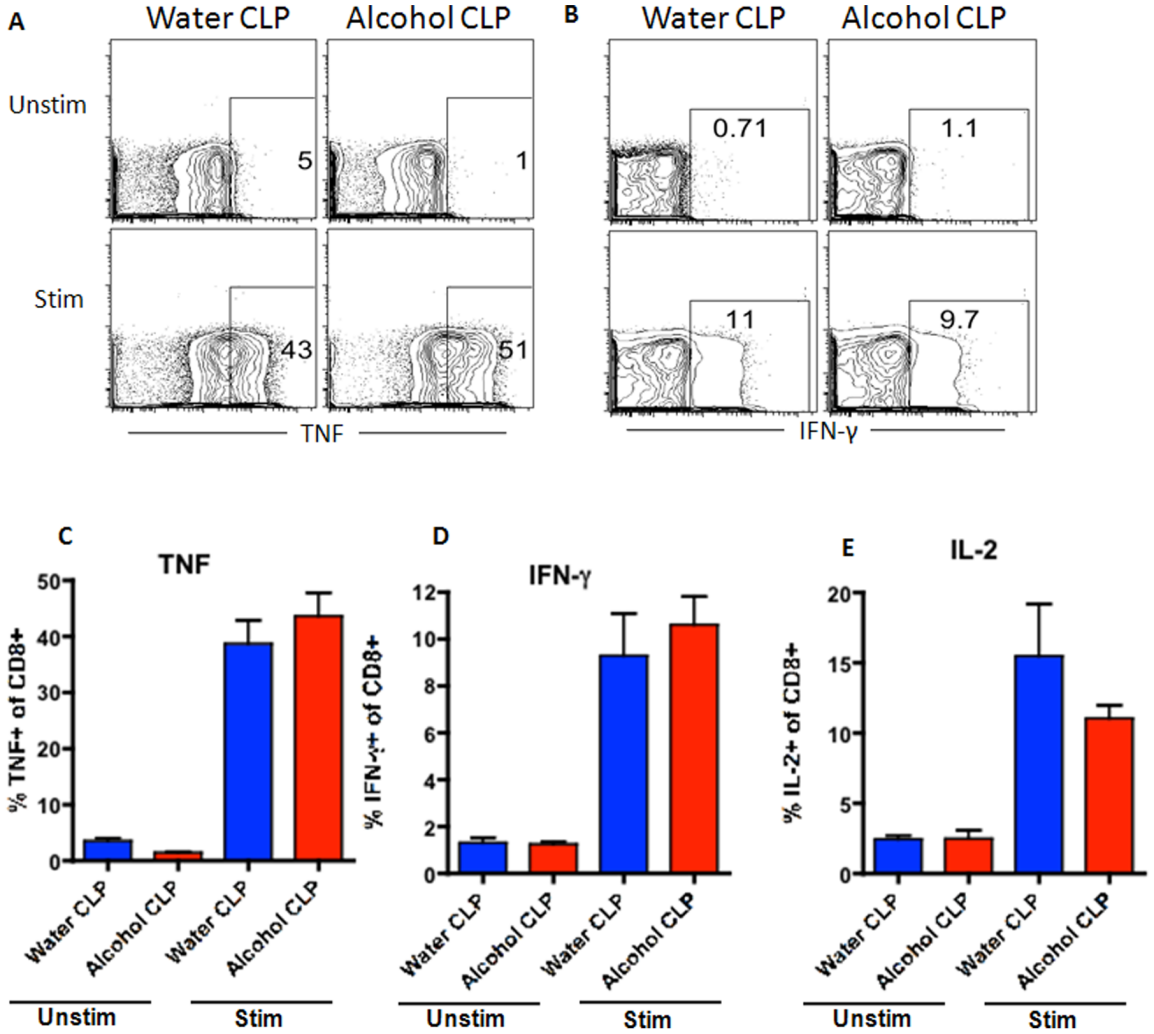
Effect of chronic alcohol ingestion on inflammatory cytokine secretion by CD8+ T cells during sepsis. Splenocytes from septic water-fed mice (n = 6/group) and septic alcohol-fed mice (n = 8/group) were harvested, restimulated with PMA/ionomycin for 18 h *ex vivo*, and stained intracellularly for the presence of inflammatory cytokines. The frequencies of TNF-secreting (A, C), IFN-γ-secreting (B, D), and IL-2-secreting (E) CD8+ T cells were unchanged between septic alcohol-fed mice and septic water-fed mice.

**Figure 11:**
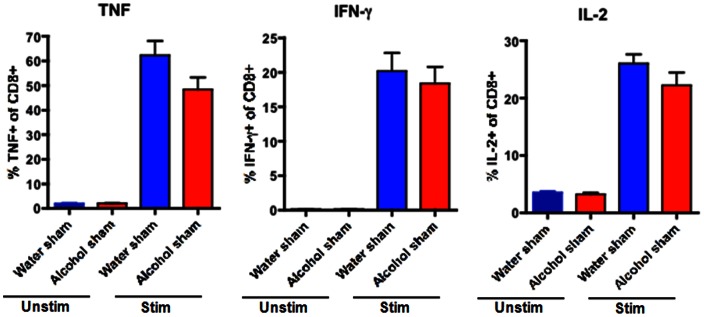
Effect of chronic alcohol ingestion on inflammatory cytokine secretion by CD8+ T cells in sham mice. Splenocytes from sham water-fed mice (n = 5/group) and sham alcohol-fed mice (n = 5/group) were harvested, restimulated with PMA/ionomycin for 18 h *ex vivo*, and stained intracellularly for the presence of inflammatory cytokines. The frequencies of TNF-secreting, IFN-γ-secreting, and IL-2-secreting CD8+ T cells were unchanged between sham alcohol-fed mice and sham water-fed mice.

Similar to CD4+ cells, CD8+ cells comprised a similar frequency of total lymphocytes in the spleen of both septic alcohol-fed and septic water-fed mice (Figure 6B). This was also true for sham alcohol-fed and sham water-fed mice (Figure 7B). However, in contrast to CD4+ cells, there were no differences in TNF, IFN-γ or IL-2 production in splenic CD8+ cells isolated from septic alcohol-fed mice as compared to septic water-fed mice following *ex vivo* stimulation (Figure 10). Similarly, no differences in CD8+ T cell cytokine secretion were noted in sham alcohol-fed and sham water-fed mice (Figure 11).

**Figure 12:**
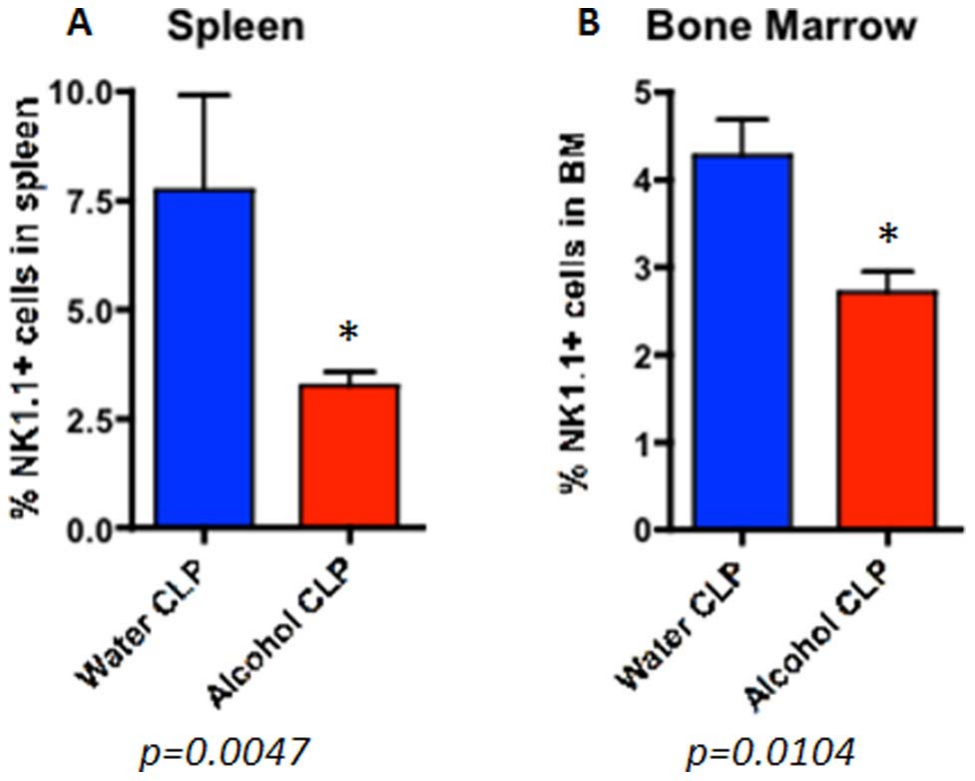
Effect of chronic alcohol ingestion on the size of NK cell compartment in the spleen and bone marrow during sepsis. Septic alcohol-fed mice (n = 8/group) had significantly reduced frequencies of NK1.1+ NK cells in both the (A) spleen (p = 0.005) and (B) BM (p = 0.01) as compared to septic water-fed mice (n = 8/group).

**Figure 13:**
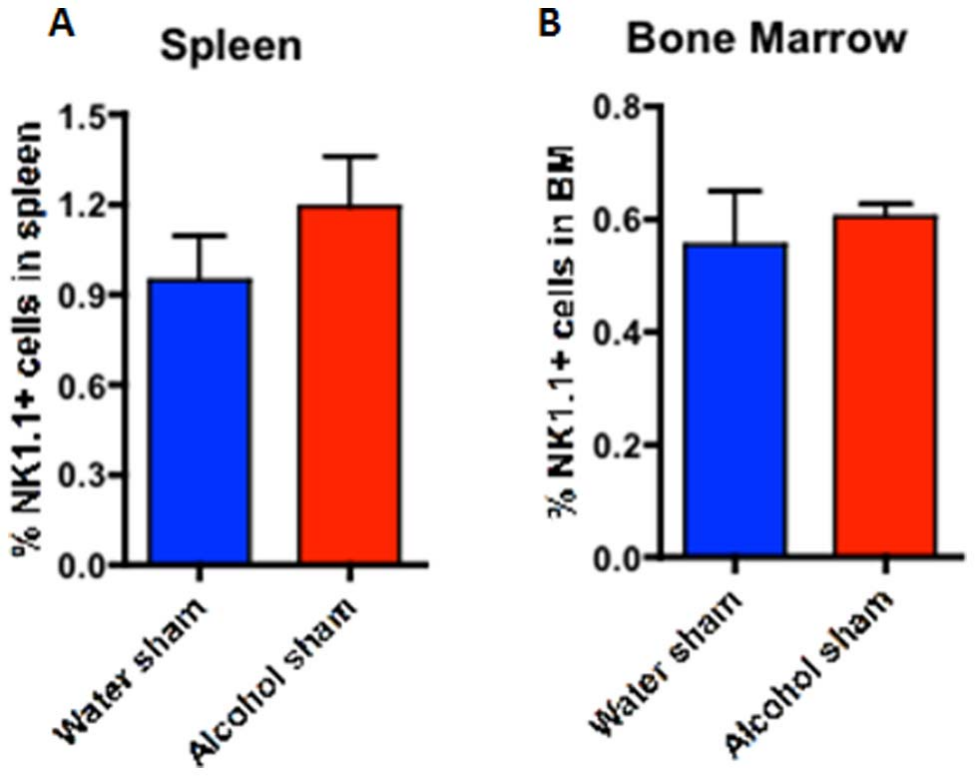
Effect of chronic alcohol ingestion on the size of NK cell compartment in the spleen and bone marrow in sham mice. Sham alcohol-fed mice (n = 5/group) had similar frequencies of NK1.1+ NK cells in both the (A) spleen and (B) BM as compared to sham water-fed mice (n = 5/group).

Whereas the frequencies of CD4+ and CD8+ cells did not differ following chronic alcohol ingestion and sepsis, the frequency of NK cells was lower in both the spleen and bone marrow in septic alcohol-fed mice than septic water-fed mice (Figure 12). Of note, this difference was specific to the setting of sepsis as no differences in NK cell frequencies in either the spleen or bone marrow were observed in sham alcohol-fed as compared to sham water-fed mice (Figure 13). Furthermore, no differences in splenic histology were detected between septic alcohol-fed mice and septic water-fed mice (data not shown). Serum cytokines were generally similar between septic alcohol-fed mice and septic water-fed mice (Table 1) although serum IL-6 was lower in the septic alcohol-fed mice.

**Table 1:** Serum Cytokines.

Cytokines (pg/ml)	Water (n = 7–9)	Alcohol (n = 7–9)	P Value
IL-1β	9096 ± 1947	7630 ± 1262	ns
IL-6	22421 ± 3752	11872 ± 2220	0.04
IL-10	2203 ± 521	3660 ± 849	ns
IL-13	1415 ± 204	1600 ± 529	ns
G-CSF	306011 ± 6606	312684 ± 1580	ns
IFN-γ	22 ± 3	17 ± 1	ns
MCP-1	42164 ± 7906	29871 ± 5586	ns
TNF-α	667 ± 98	984 ± 194	ns

### Effect of chronic ethanol ingestion on lungs following sepsis

**Figure 14:**
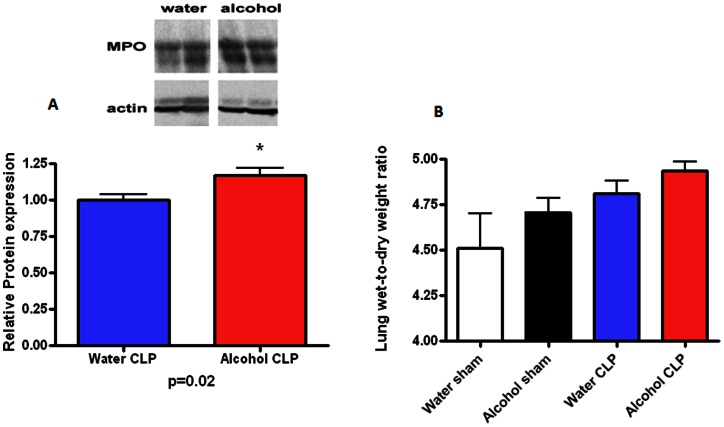
Effect of chronic alcohol ingestion on pulmonary myeloperoxidase expression and lung wet-to-dry ratio. Protein levels of myeloperoxidase (A) were higher in septic alcohol-fed mice than septic water-fed mice. No statistically significant differences were noted in lung wet-to-dry ratios (B) in mice, regardless of whether they were sham or 24 hours after CLP, and regardless of whether they were water-fed or alcohol-fed (n = 5-10/group).

Pulmonary MPO levels were higher in septic alcohol-fed mice than septic water-fed mice (Figure 14A, p = 0.02). BAL levels of G-CSF and TGF-β were also higher in septic alcohol-fed mice than septic water-fed mice although there were not significant differences in IL-1 β, IL-6, IL-10, IL-13, IFN-γ, MCP-1 or TNF levels (Table 2). No differences in wet to dry ratio were identified between alcohol-fed mice and water-fed mice, regardless of whether they were septic (Figure 14B). Pulmonary histology was also similar between septic alcohol-fed mice and septic water-fed mice (data not shown).

**Table 2:** BAL Cytokines.

Cytokines (pg/ml)	Water (n = 7–9)	Alcohol (n = 7–9)	P Value
IL-1β	1280 ± 302	1768 ± 602	ns
IL-6	99 ± 23	213 ± 93	ns
IL-10	232 ± 41	186 ± 41	ns
IL-13	911 ± 407	1729 ± 999	ns
G-CSF	17280 ± 3114	33976 ± 8226	0.03
IFN-γ	35 ± 11	39 ± 5	ns
MCP-1	808 ± 268	1065 ± 281	ns
TNF-α	159 ± 41	308 ± 76	ns
TGF-β	376 ± 81	778 ± 71	0.004

### Effect of chronic ethanol ingestion on bacteremia, local host response and liver pathology following sepsis

Trace amounts of bacteria (maximal 60 cfu/ml) was identified in 4/9 alcohol-fed mice whereas all 8 water-fed mice sampled were sterile (p = ns). Peritoneal fluid cytokines were generally similar between septic alcohol-fed mice and septic water-fed mice (Table 3) although peritoneal fluid MCP-1 was higher in the septic alcohol-fed mice. Liver histology was also similar between septic alcohol-fed mice and septic water-fed mice (data not shown).

**Table 3:** Peritoneal Fluid Cytokines.

Cytokines (pg/ml)	Water (n = 7–9)	Alcohol (n = 7–9)	P Value
IL-1β	3253 ± 1467	1351 ± 724	ns
IL-6	16502 ± 4876	10774 ± 1899	ns
IL-10	816 ± 264	501 ± 85	ns
IL-13	1207 ± 552	619 ± 126	ns
G-CSF	280086 ± 26961	250883 ± 21230	ns
IFN-γ	9 ± 3	11 ± 4	ns
MCP-1	24145 ± 5434	55975 ± 7518	0.007
TNF-α	524 ± 362	119 ± 19	ns

### Effect of chronic ethanol ingestion on pancreatic metabolomics following sepsis

**Figure 15:**
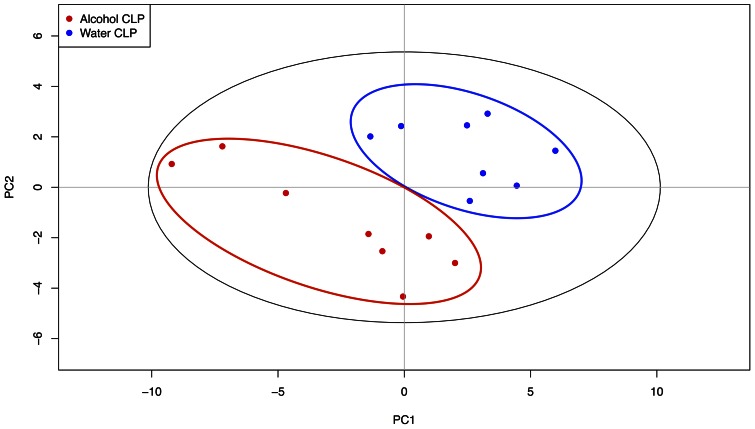
Effect of chronic alcohol ingestion on pancreatic metabolite-based differentiation. PLS-DA scores plot showed pancreas metabolite-based differentiation of septic alcohol-fed and septic water-fed mice. Metabolic profiles obtained via ^1^H NMR spectroscopy were used to construct the PLS-DA model. Points on the scores plot correspond to pancreatic tissue samples obtained from either septic alcohol-fed mice (red) or septic water-fed mice (blue). Cross-validation of the model demonstrated that septic alcohol-fed mice can be reliably differentiated from septic water-fed mice based upon pancreas metabolic profiles (R^2^ = 0.92, Q^2^ = 0.65).

**Figure 16:**
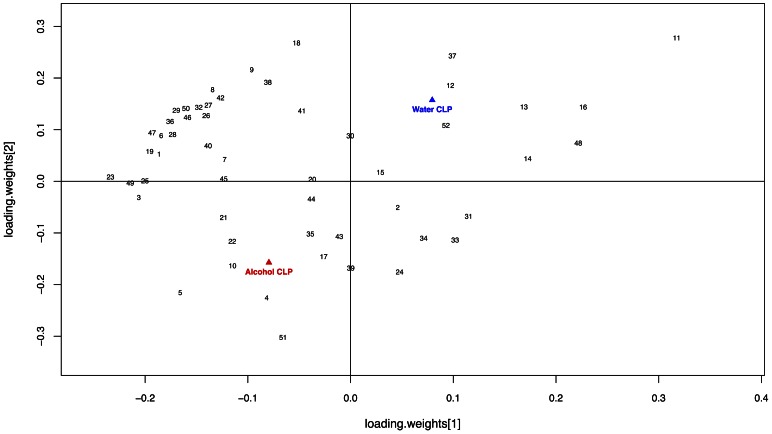
Effect of chronic alcohol ingestion on pancreatic metabolite concentration. PLS-DA loadings plot of profiled metabolites obtained from septic alcohol-fed and water-fed mice. Metabolites that contribute most to differences observed between the two experimental groups occupy the lower left quadrant of the plot (alcohol-fed) or in the upper right quadrant of the plot (water-fed). Metabolites important to the differentiation of pancreatic tissue include 11 (Choline), 16 (Fumarate), 48 (Uracil), 13 (Creatine Phosphate), 14 (Cytidine), 37 (Phthalate), 12 (Creatine), 52 (sn-Glycero-3-phosphocholine; water-fed > alcohol-fed) as well as 5 (Adenosine), 51 (Xanthine), 4, (Acetoacetate), 21 (Glycine), 22 (Guanidoacetate), 3 (Acetate), and 10 (Betaine; alcohol-fed > water-fed). The full list of metabolites examined is available as Table S1.

A PLS-DA model was constructed to evaluate the metabolic differences between pancreatic tissue from septic alcohol-fed mice and septic water-fed animals during sepsis (Figure 15). The model optimized separation of samples according to treatment group. The model was described by fit parameters R^2^ = 0.92 and Q^2^ = 0.65, indicating both a good fit to the data and good predictive value. The corresponding loadings plot (Figure 16) revealed increases in concentrations of adenosine, xanthine, acetoacetate, guanidoacetate, acetate, betaine, and 3-hyroxybutyrate for septic alcohol-fed mice relative to septic water-fed mice. Increased concentrations of metabolites in the septic water-fed mice relative to septic alcohol-fed mice included choline, fumarate, uracil, cytidine, creatine, and creatine phosphate. Despite differences in pancreatic metabolomics, there were no differences in pancreatic histology between septic alcohol-fed mice and septic water-fed mice (data not shown).

## Discussion

Patients with alcohol use disorders who become septic have worse outcomes than other septic patients. Our mouse model of chronic alcohol ingestion followed by sepsis, which shows that alcohol-fed animals have a significantly higher mortality following CLP than water-fed animals, thus mimics the clinical scenario. Our results demonstrate a number of associated abnormalities that may play a role in this differing mortality.

In isolation, both alcohol and sepsis have been shown to alter intestinal integrity to varying degrees. Although our results did not demonstrate a statistically significant difference between sham alcohol-fed mice and sham water-fed mice, alcohol has previously been shown to induce apoptosis in human intestinal cells *in vitro* [32]. Additionally, chronic alcohol usage has been shown to increase intestinal proliferation leading to hyperplasia [33], [34], although the opposite effect has been seen in pregnancy where proliferation is decreased [35]. Sepsis also induces gut epithelial apoptosis in both animal models and human autopsy studies of sepsis [24], [36]–[40] and prevention of gut epithelial apoptosis in animal models improves survival in both CLP and pneumonia [26], [28], [38], [39], [41], [42]. In addition, proliferation is decreased and villus length is decreased in preclinical models of critical illness [36], [41], [43]–[45].

Our results showed that intestinal integrity was significantly worsened by the combination of chronic alcohol ingestion and sepsis, more than could have been predicted from examining either variable in isolation. Since the gut is hypothesized to be the “motor” of the systemic inflammatory response syndrome [46]–[50], the exacerbated injury induced in alcohol-fed mice, may have played a role in the increased mortality from sepsis, although this remains to be tested. One possible mechanism through which altered gut integrity could impact mortality is via alterations in intestinal permeability.

Similar to other elements of intestinal integrity, gut permeability has been shown to be altered by both alcohol and sepsis in isolation. Although our results failed to show a statistically significant difference in permeability between sham alcohol-fed mice and sham water-fed mice, alcohol abuse has been shown by other groups to increase gut permeability in the small intestine [51]–[54]. Similar findings have been found with acute alcohol intoxication in the context of burn and trauma [55], [56]. Although the mechanisms behind this are only partially understood, alterations in the tight junction can lead to intestinal hyperpermeability, which can lead to translocation of bacteria or bacterial products from the gut lumen [57], [58]. Additionally, intestinal barrier function is decreased in critical illness [59]–[62], and it has been proposed that increased intestinal permeability perpetuates systemic inflammation [63]. Our data demonstrated a synergistic worsening of intestinal barrier function from the combination of chronic alcohol ingestion and sepsis. This increased permeability was associated with increases in occludin and ZO-1 in alcohol-fed septic mice compared to water-fed septic mice. This suggests that the alcohol/sepsis combination increases permeability through a different mechanism than sepsis alone, in light of the fact that we have previously shown that CLP does not upregulate either occludin or ZO-1 [26].

The adaptive immune response is also impacted by both alcohol and sepsis in isolation; however, our results appear to show an interplay between them that could not have been predicted from examining each variable in isolation, as we did not observe any difference in the frequencies of immune cell subset frequencies or functionality in the setting of chronic alcohol consumption alone. In contrast, sepsis induces a marked decrease in CD4+ and CD8+ lymphocytes secondary to massive apoptosis in these cell types [64]–[67]. Our results demonstrate that the increased mortality seen in septic mice following chronic alcohol ingestion is not related to a further reduction in CD4+ or CD8+ cells, as these were similar between septic alcohol-fed and water-fed mice. However, we observed a significant upregulation in the production of TNF and IFN-γ in CD4+ (but not CD8+) T cells in septic alcohol-fed mice compared to septic water-fed mice. Because splenocytes have been shown to exhibit decreased production of TNF and IFN-γ in non-alcoholic septic patients [68], this suggests that a more robust early pro-inflammatory response in mice with chronic alcohol ingestion may be maladaptive following sepsis. In addition, these data underscore that significant differences may exist between the local inflammatory response and that seen in systemic cytokines (compare figure 7 to table 1).

It has also been suggested that production of inflammatory cytokines by CD4+ T cells may be increased but production of these same cytokines by other cell types including inflammatory monocytes, macrophages, and endothelial cells may be reduced [19]. These results are also complementary to *in vitro* work demonstrating that increasing alcohol concentrations are synergistic to bacteria in production of proinflammatory cytokines [69].

We also found that the frequency of splenic and bone marrow NK cells was lower in septic alcohol-fed mice as compared to septic water-fed mice. Importantly, this effect was dependent on the combination of chronic alcohol ingestion and sepsis, as no differences in NK cell frequencies were observed between sham alcohol-fed and sham water-fed mice in our study. While it has previously been reported that chronic alcohol consumption can induce abnormalities in NK cell activity and subset distribution in both humans and mice, at least in part via compromised NK cell release from the bone marrow [70], these alterations have been shown to be a function of the duration of alcohol consumption. For example, one study found that the initially decreased frequencies of NK cells in alcohol-fed mice had normalized by 2 weeks of alcohol ingestion, a finding which is consistent with our data [71]. Thus, we have identified an effect of chronic ethanol consumption on NK cell frequencies that is precipitated in the setting of sepsis. A similar decrease in splenic NK cells is also induced by sepsis in the absence of alcohol consumption [64], [72]. The significance of both the difference in NK frequencies and their functional capacity under these conditions remains to be determined.

There is significant evidence that chronic alcohol ingestion impacts the lungs by causing oxidative stress and alveolar epithelial barrier dysfunction [73]–[76]. Resident alveolar macrophages are impaired by chronic alcohol abuse, leading to a decrease in their ability to phagocytose bacteria, and release cytokines and chemokines responsible for bacterial killing [77]–[79]. This is secondary to a defect in GM-CSF signaling necessary for macrophage maturation and function and can be reversed in animals with recombinant GM-CSF [80], [81]. The lung is also one of the most commonly affected organs in critical illness, with 75,000 deaths annually in the United States from ARDS [82], [83]. Additionally, sepsis is a leading cause of ARDS [84] although the impact of respiratory failure on mortality from sepsis remains unclear. Similarly, while CLP is a frequently used model of acute lung injury [85], a recent study shows that CLP does not induce lung injury in outbred female mice, even in an injury associated with 100% mortality [86].

Our results demonstrated that pulmonary MPO levels and BAL levels of GCS-F and TGF-β were higher in septic alcohol-fed mice compared to septic water-fed mice. In contrast, lung wet to dry ratio (a surrogate for pulmonary edema) and histology were similar between the animals as were other BAL cytokines. This expands the relatively limited data on the role of chronic alcohol ingestion in the setting of extrapulmonary sepsis. It has previously been shown using a similar model to that used herein that rats had lower glutathione levels and fluid protein levels in lung lavage fluid as well as worsened hypoxemia [23]. Additional studies have examined CLP in the setting of acute alcohol intoxication [87] or twenty four hours after trauma/hemorrhage following acute alcohol intoxication [88]. The former study demonstrated that mice given a 10 hour alcohol infusion had increased lung neutrophil infiltration while the latter showed increased mortality in mice given alcohol followed by hemorrhage followed by CLP. The clinical utility of studying acute alcohol intoxication in the setting of CLP is questionable, however, as this mimics an unlikely clinical scenario, since unlike burn or trauma (which are common after acute alcohol intoxication), it is uncommon for a patient to develop peritonitis after a one-time drinking episode or to develop peritonitis one day after a trauma after a one-time drinking binge.

Chronic alcohol usage is associated with pancreatitis. Although there were no histologic differences in pancreatic tissue between septic alcohol-fed and septic water-fed mice, a pancreatic metabolomics evaluation [30] was undertaken which demonstrated significant differences between the groups. This complements existing studies on serum, liver and urine metabolomics in rodents with chronic alcohol ingestion [89], [90] as well as serum and BAL metabolomics following CLP [91], [92]. Since we did not perform baseline proteomic analyses in either sham alcohol-fed mice or sham water-fed mice, it is difficult to know which of the metabolomic differences seen were due to the effects of chronic alcohol ingestion, sepsis in isolation or a combination. Nonetheless, these results may be the starting point of future studies examining this in more detail.

This study has a number of limitations. First, it is not clear from our studies which phenotypic differences between septic alcohol-fed and septic water-fed animals were responsible for the difference in mortality and which were associative but non-causal. Similarly, the significance of some of our findings (decreased NK cells in septic alcohol-fed mice, for instance) is unclear, without additional functional studies. Next, all non-survival experiments were performed at a single timepoint, 24 hours. Assaying animals at different timepoints would likely have led to additional insights that were missed by our design. Finally, the most common mouse strain for both chronic alcohol and sepsis studies is C57BL/6, and how strain impacts our results is unknown.

Despite these limitations, we have demonstrated that chronic alcohol ingestion leads to a significant increase in mortality from sepsis. This is associated with changes in a number of organ systems, including alterations in gut integrity, function of CD4+ T cells and the lungs. Performing functional studies to elucidate the differences between septic alcohol-fed and septic water-fed mice may yield insights that eventually allow targeted treatment of a large group of patients with increased morbidity and mortality in the ICU.

## Supporting Information

Table S1 Metabolites corresponding to each number in Figure 16.
(DOC)
