# Correction: Chronic Alcohol Ingestion Increases Mortality and Organ Injury in a Murine Model of Septic Peritonitis

**DOI:** 10.1371/journal.pone.0239568

**Published:** 2020-09-17

**Authors:** Benyam P. Yoseph, Elise Breed, Christian E. Overgaard, Christina J. Ward, Zhe Liang, Maylene E. Wagener, Daniel R. Lexcen, Elizabeth R. Lusczek, Greg J. Beilman, Eileen M. Burd, Alton B. Farris, David M. Guidot, Michael Koval, Mandy L. Ford, Craig M. Coopersmith

After publication of this article [1], concerns were raised about the following results in Fig 5:

- β-actin data shown in the Water (lanes 1, 2) and Alcohol (lane 1) panels in Fig 5B appear similar to β-actin data shown in the Water (lanes 1, 2) and Alcohol (lane 2) panels, respectively, of Fig 5C.
- In Fig 5D, Claudin-2 panel, the same data appear to be presented for the Water and Alcohol groups.

**Fig 5:**
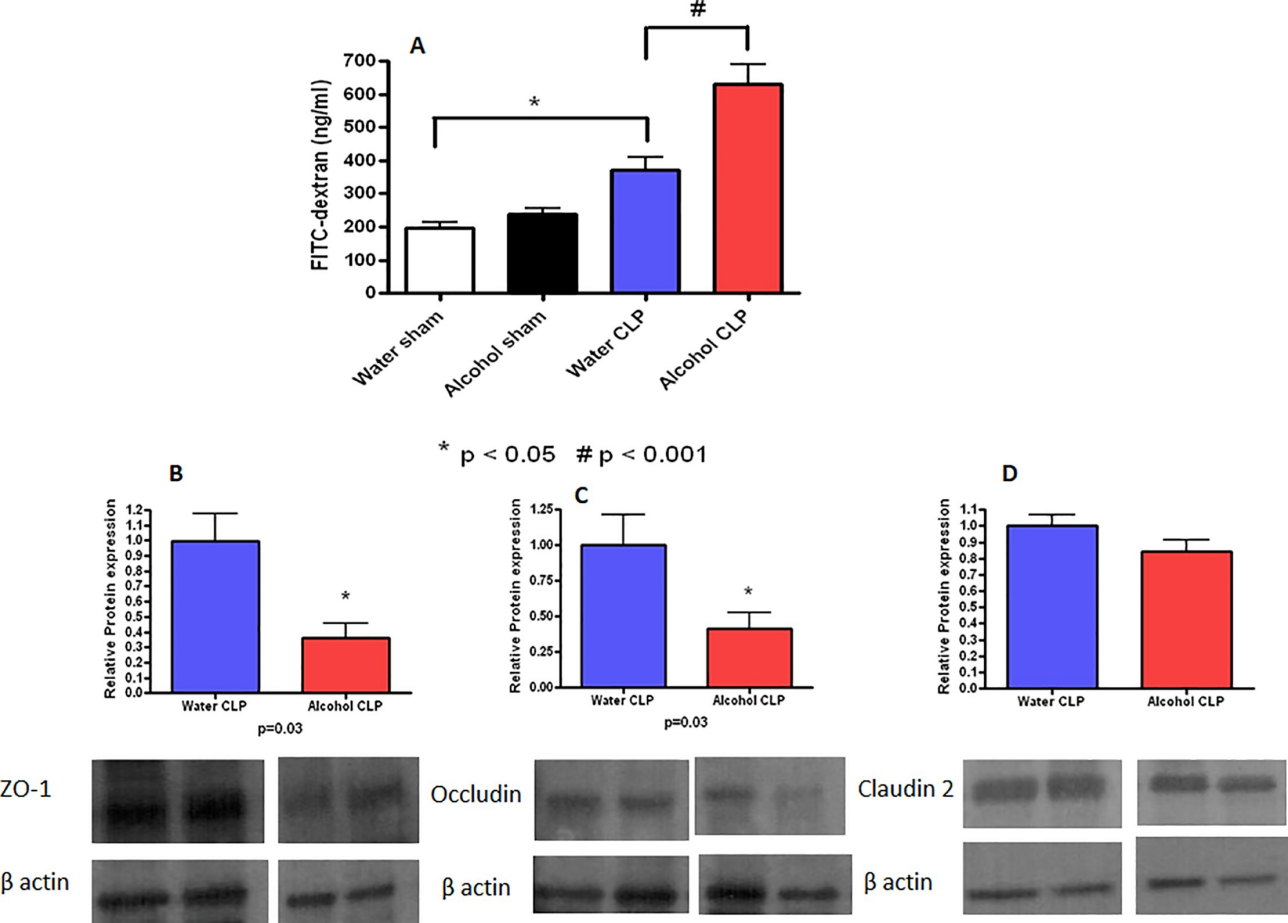
Effect of chronic alcohol ingestion on intestinal permeability. Chronic alcohol ingestion did not impact permeability (A) in sham mice compared to water-fed mice (n = 10/group). Septic water-fed mice (n = 13/group) had hyperpermeability compared to sham water-fed mice. Septic alcohol-fed mice (n = 15/group) had a further increase in intestinal permeability compared to septic water-fed mice. Protein levels of ZO-1 (B) and occludin (C) were lower in septic alcohol-fed mice compared to septic water-fed mice although claudin-2 levels (D) were similar. Representative blots for each tight junction protein are depicted; densitometry was determined by normalizing expression to β-actin.

The authors noted that an error was made in preparing the Claudin-2 panel for the Alcohol group in Fig 5D.

Regarding the β-actin duplications, as shown in S1 File, the duplicated β-actin data and corresponding ZO-1 and Occludin data correspond to the same lanes in all three blots. For other experiments in the same series, laboratory records indicate that the authors consistently ran the same protein samples on parallel blots probed with ZO-1, Occludin, and β-actin. Thus, the authors noted that the same β-actin control data likely apply to ZO-1 and Occludin experiments reported in Fig 5B and 5C. The authors apologize that this aspect of the experimental design was not clearly described in the original article.

A corrected version of Fig 5 is provided here. The full-sized original blots are no longer available, but images showing all experimental lanes of the original blots are provided in S1 File, and the raw quantification data are in S2 File.

Raw data are available for all other data in the manuscript except source flow cytometry plots for Figs 8 and 10.

The authors apologize for the errors in the published article.

## Supporting information

S1 File Pictures of original Western blots. Western blot data supporting Fig 5. For each blot, lanes 1–5 are water CLP samples, lane 6 is blank, and lanes 7–12 are alcohol-CLP samples.
(TIF)

S2 File TJ proteins EtOH vs H2O prism file. Prism file containing raw data of the majority of the data in the manuscript including Figs 1–5, 8–11 and 14.
(PZF)
