# Supplementary material for: Chronic Alcohol Ingestion Increases Mortality and Organ Injury in a Murine Model of Septic Peritonitis
Source: PLoS One. 2013 May 22;8(5):e62792. doi: 10.1371/journal.pone.0062792 (PMC3661585; doi:10.1371/journal.pone.0062792)
Supplement: Table S1 — Metabolites corresponding to each number in Figure 16. (DOC) [file pone.0062792.s001.doc]

| **Metabolite** | **Number** |
| --- | --- |
| 3-Hydroxybutyrate | **1** |
| ATP | **2** |
| Acetate | **3** |
| Acetoacetate | **4** |
| Adenosine | **5** |
| Alanine | **6** |
| Arginine | **7** |
| Asparagine | **8** |
| Aspartate | **9** |
| Betaine | **10** |
| Choline | **11** |
| Creatine | **12** |
| Creatine phosphate | **13** |
| Cytidine | **14** |
| Formate | **15** |
| Fumarate | **16** |
| Glucose | **17** |
| Glutamate | **18** |
| Glutamine | **19** |
| Glycerol | **20** |
| Glycine | **21** |
| Guanidoacetate | **22** |
| Histidine | **23** |
| Hypoxanthine | **24** |
| Inosine | **25** |
| Isoleucine | **26** |
| Lactate | **27** |
| Leucine | **28** |
| Lysine | **29** |
| Malonate | **30** |
| Mannose | **31** |
| Methionine | **32** |
| Niacinamide | **33** |
| O-Phosphocholine | **34** |
| Oxypurinol | **35** |
| Phenylalanine | **36** |
| Phthalate | **37** |
| Proline | **38** |
| Putrescine | **39** |
| Pyruvate | **40** |
| Ribose | **41** |
| Serine | **42** |
| Succinate | **43** |
| Taurine | **44** |
| Trimethylamine N-oxide | **45** |
| Tryptophan | **46** |
| Tyrosine | **47** |
| Uracil | **48** |
| Uridine | **49** |
| Valine | **50** |
| Xanthine | **51** |
| sn-Glycero-3-phosphocholine | **52** |
