# Supplementary figures and images for: Correction: Chronic Alcohol Ingestion Increases Mortality and Organ Injury in a Murine Model of Septic Peritonitis
Source: PLoS One. 2020 Sep 17;15(9):e0239568. doi: 10.1371/journal.pone.0239568 (PMC7498101; doi:10.1371/journal.pone.0239568)

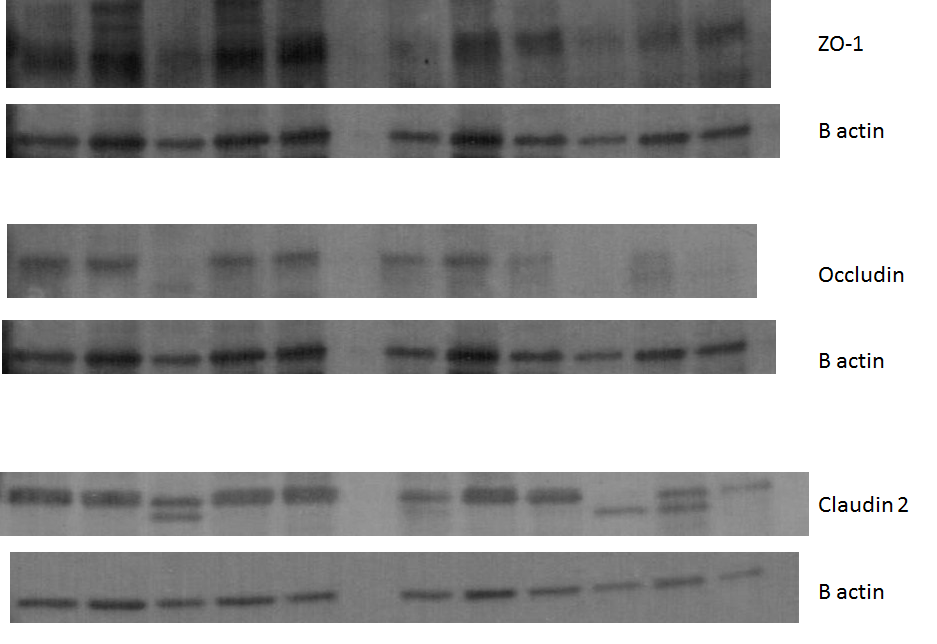

Supplement: S1 File — Western blot data supporting Fig 5. For each blot, lanes 1–5 are water CLP samples, lane 6 is blank, and lanes 7–12 are alcohol-CLP samples. (TIF) [file pone.0239568.s001.tif]
